# Supplementary figures and images for: Temozolomide-Acquired Resistance Is Associated with Modulation of the Integrin Repertoire in Glioblastoma, Impact of α5β1 Integrin
Source: Cancers (Basel). 2022 Jan 12;14(2):369. doi: 10.3390/cancers14020369 (PMC8773618; doi:10.3390/cancers14020369)

Sup. Figure S1

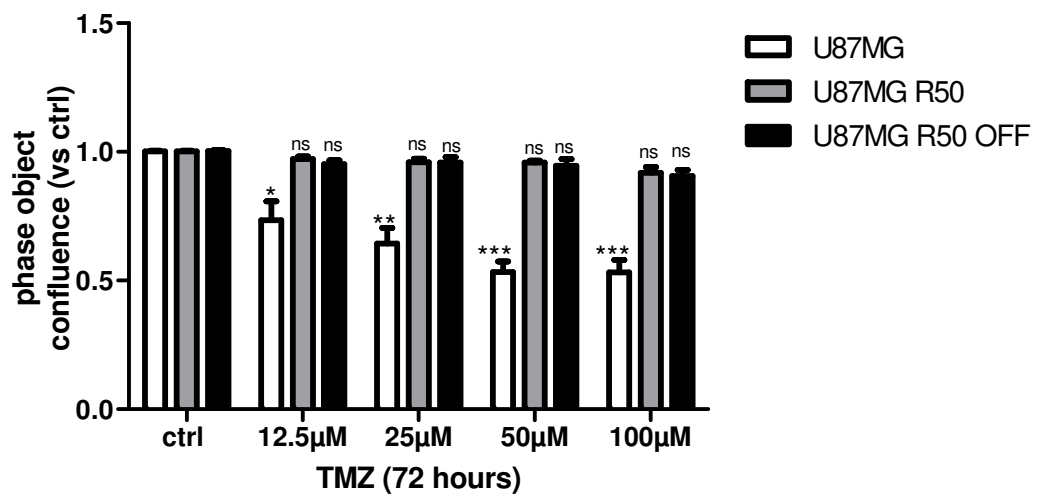

A

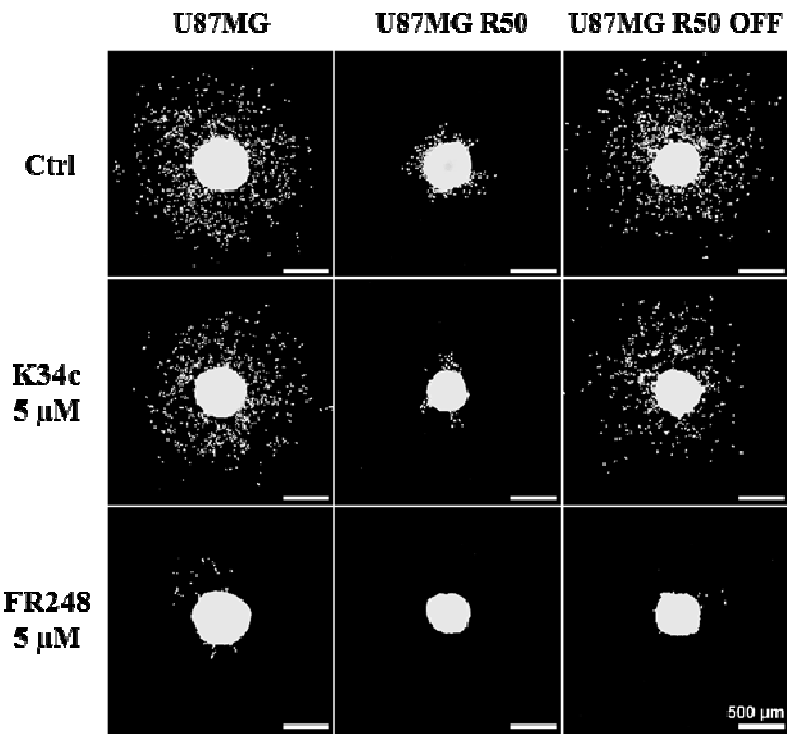

B

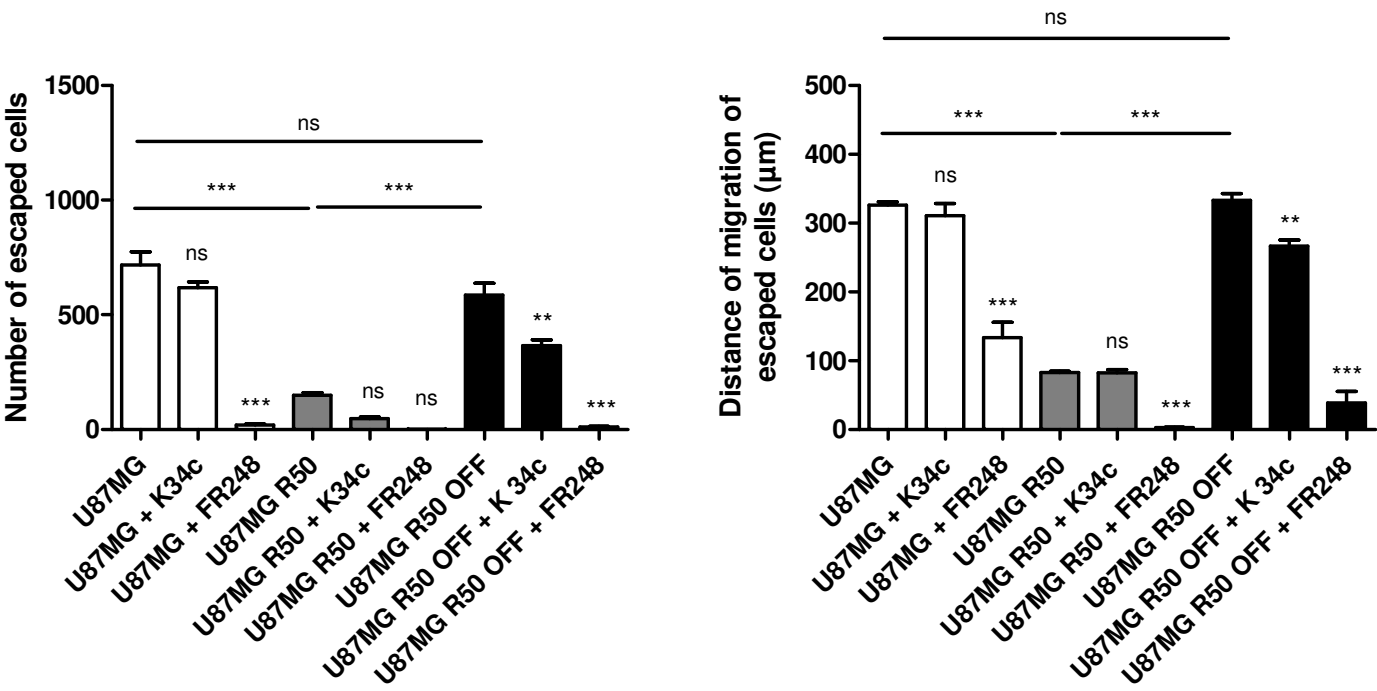

Sup. Figure S3

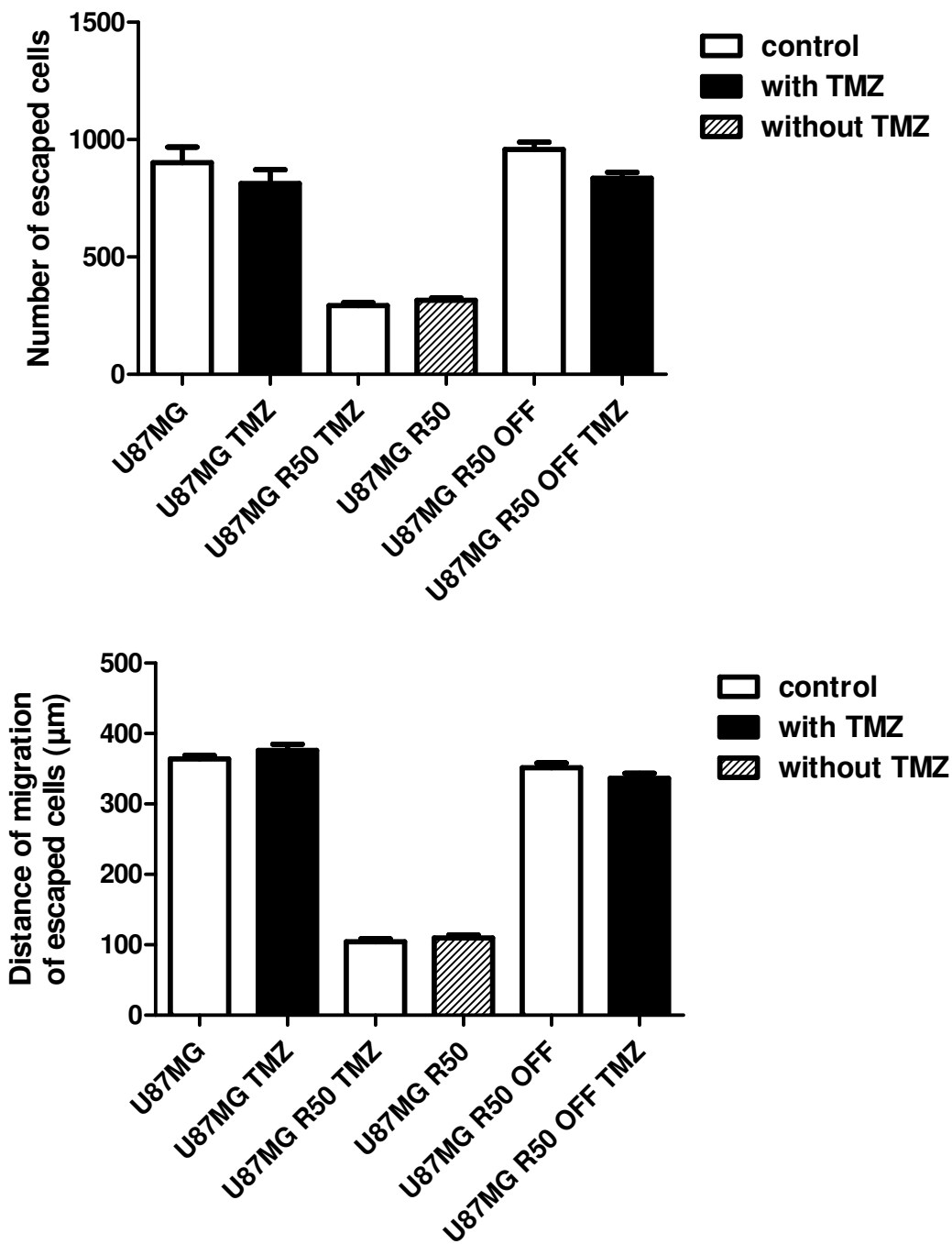

A

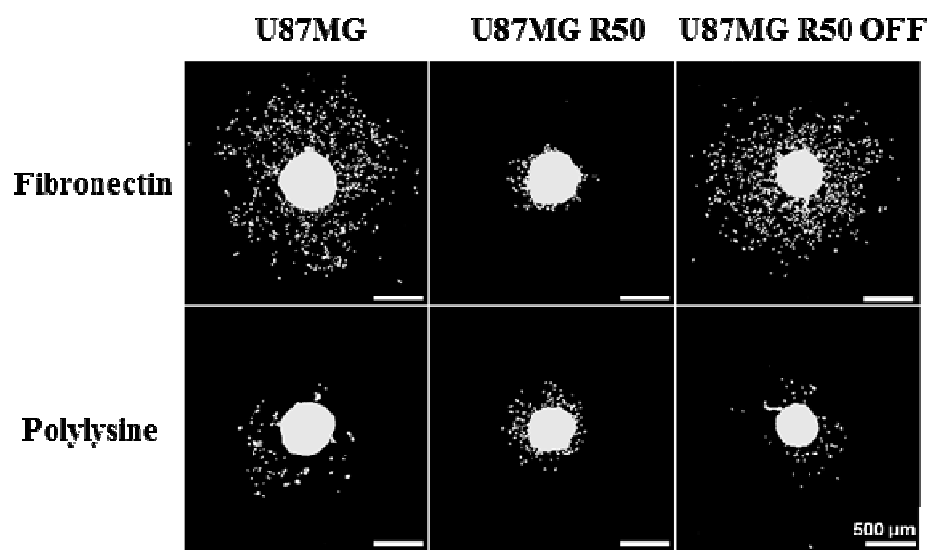

B

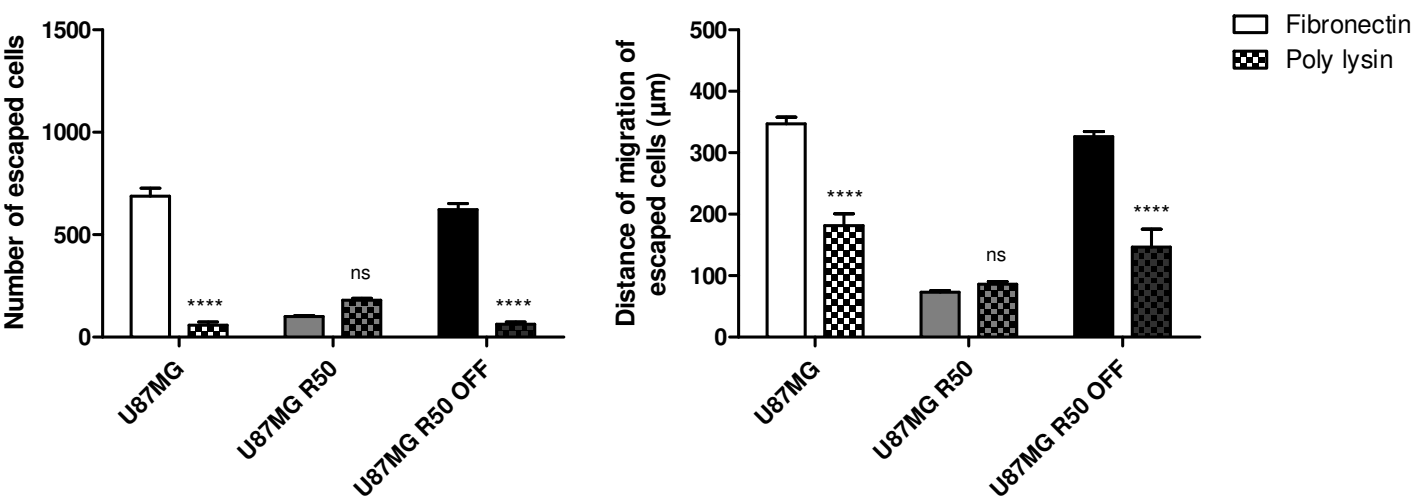

Sup. Figure S5

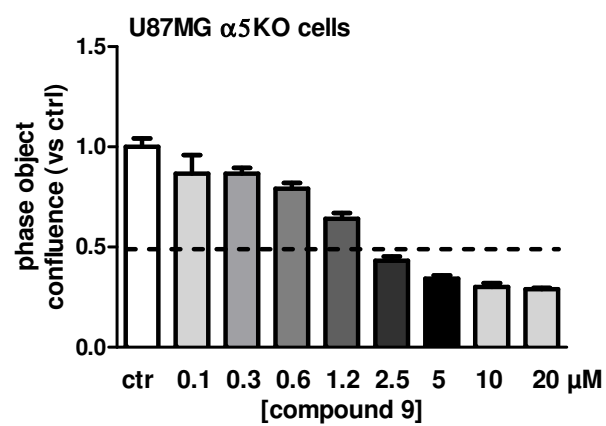

Supplement: Supplementary file 1 [file cancers-14-00369-s001.zip › cancers-1456579-supplementary.pdf]
